# Supplementary material for: Integrated Analysis of the Lung Microbiome and Metabolome Reveals Associations Between Amino Acid Metabolism and Pulmonary Fibrosis in a Bleomycin-Induced Mouse Model
Source: Int J Mol Sci. 2026 Jun 30;27(13):5895. doi: 10.3390/ijms27135895 (PMC13362081; doi:10.3390/ijms27135895)
Supplement: Supplementary file 1 [file ijms-27-05895-s001.zip › result/4.MetDiffAnalysis/Heatmap_diff/Diff_Heatmap_all.detail.pdf]

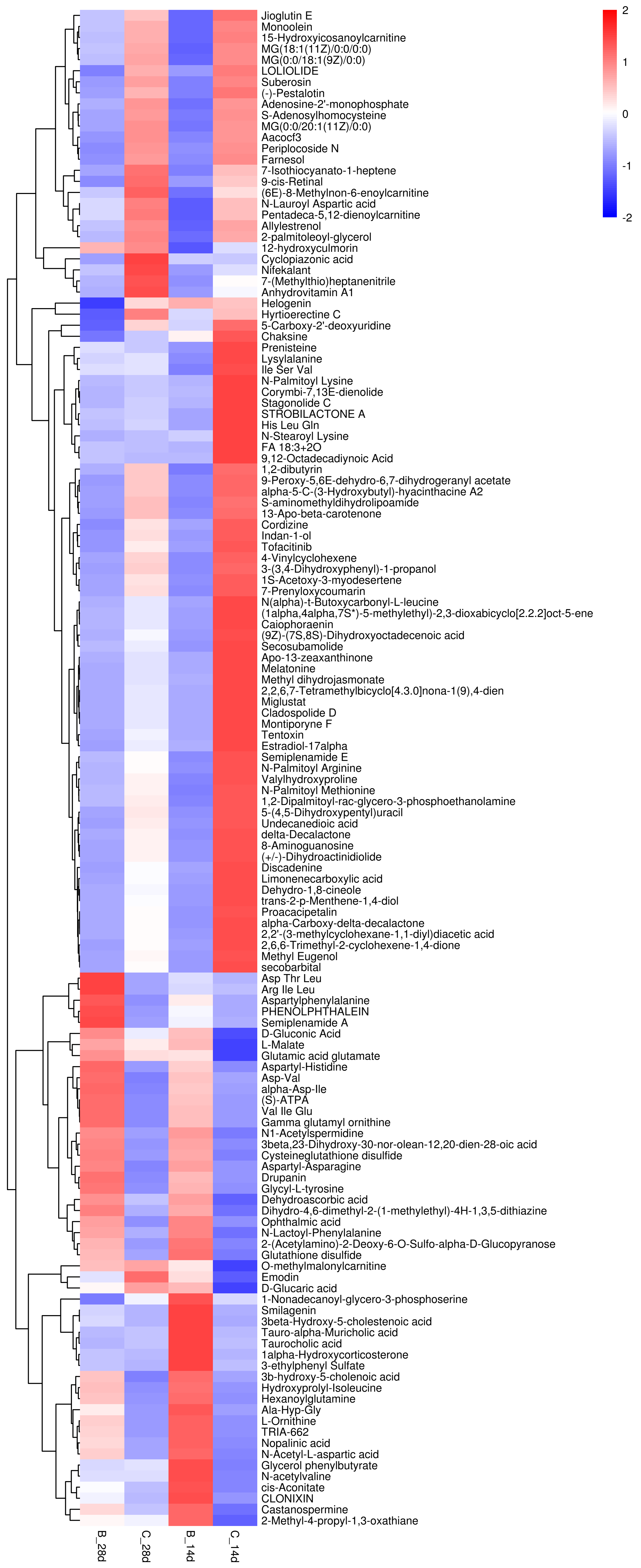

B_28d
C_28d
B_14d
C_14d
2
1
0
-1
-2
Jioglutin E
Monoolein
15-Hydroxyicosanoylcarnitine
MG(18:1(11Z)/0:0/0:0)
MG(0:0/18:1(9Z)/0:0)
LOLIOLIDE
Suberosin
(-)-Pestalotin
Adenosine-2'-monophosphate
S-Adenosylhomocysteine
MG(0:0/20:1(11Z)/0:0)
Aacocf3
Periplocoside N
Farnesol
7-Isothiocyanato-1-heptene
9-cis-Retinal
(6E)-8-Methylnon-6-enoylcarnitine
N-Lauroyl Aspartic acid
Pentadeca-5,12-dienoylcarnitine
Allylestrenol
2-palmitoleoyl-glycerol
12-hydroxyculmorin
Cyclopiazonic acid
Nifekalant
7-(Methylthio)heptanenitrile
Anhydrovitamin A1
Helogenin
Hyrtioerectine C
5-Carboxy-2'-deoxyuridine
Chaksine
Prenisteine
Lysylalanine
Ile Ser Val
N-Palmitoyl Lysine
Corymbi-7,13E-dienolide
Stagonolide C
STROBILACTONE A
His Leu Gln
N-Stearoyl Lysine
FA 18:3+2O
9,12-Octadecadiynoic Acid
1,2-dibutyrin
9-Peroxy-5,6E-dehydro-6,7-dihydrogeranyl acetate
alpha-5-C-(3-Hydroxybutyl)-hyacinthacine A2
S-aminomethyldihydrolipoamide
13-Apo-beta-carotenone
Cordizine
Indan-1-ol
Tofacitinib
4-Vinylcyclohexene
3-(3,4-Dihydroxyphenyl)-1-propanol
1S-Acetoxy-3-myodesertene
7-Prenyloxycoumarin
N(alpha)-t-Butoxycarbonyl-L-leucine
(1alpha,4alpha,7S*)-5-methylethyl)-2,3-dioxabicyclo[2.2.2]oct-5-ene
Caiophoraenin
(9Z)-(7S,8S)-Dihydroxyoctadecenoic acid
Secosubamolide
Apo-13-zeaxanthinone
Melatonine
Methyl dihydrojasmonate
2,2,6,7-Tetramethylbicyclo[4.3.0]nona-1(9),4-dien
Miglustat
Cladospolide D
Montiporyne F
Tentoxin
Estradiol-17alpha
Semiplenamide E
N-Palmitoyl Arginine
Valylhydroxyproline
N-Palmitoyl Methionine
1,2-Dipalmitoyl-rac-glycero-3-phosphoethanolamine
5-(4,5-Dihydroxypentyl)uracil
Undecanedioic acid
delta-Decalactone
8-Aminoguanosine
(+/-)-Dihydroactinidiolide
Discadenine
Limonenecarboxylic acid
Dehydro-1,8-cineole
trans-2-p-Menthene-1,4-diol
Proacacipetalin
alpha-Carboxy-delta-decalactone
2,2'-(3-methylcyclohexane-1,1-diyl)diacetic acid
2,6,6-Trimethyl-2-cyclohexene-1,4-dione
Methyl Eugenol
secobarbital
Asp Thr Leu
Arg Ile Leu
Aspartylphenylalanine
PHENOLPHTHALEIN
Semiplenamide A
D-Gluconic Acid
L-Malate
Glutamic acid glutamate
Aspartyl-Histidine
Asp-Val
alpha-Asp-Ile
(S)-ATPA
Val Ile Glu
Gamma glutamyl ornithine
N1-Acetylspermidine
3beta,23-Dihydroxy-30-nor-olean-12,20-dien-28-oic acid
Cysteineglutathione disulfide
Aspartyl-Asparagine
Drupanin
Glycyl-L-tyrosine
Dehydroascorbic acid
Dihydro-4,6-dimethyl-2-(1-methylethyl)-4H-1,3,5-dithiazine
Ophthalmic acid
N-Lactoyl-Phenylalanine
2-(Acetylamino)-2-Deoxy-6-O-Sulfo-alpha-D-Glucopyranose
Glutathione disulfide
O-methylmalonylcarnitine
Emodin
D-Glucaric acid
1-Nonadecanoyl-glycero-3-phosphoserine
Smilagenin
3beta-Hydroxy-5-cholestenoic acid
Tauro-alpha-Muricholic acid
Taurocholic acid
1alpha-Hydroxycorticosterone
3-ethylphenyl Sulfate
3b-hydroxy-5-cholenoic acid
Hydroxyprolyl-Isoleucine
Hexanoylglutamine
Ala-Hyp-Gly
L-Ornithine
TRIA-662
Nopalinic acid
N-Acetyl-L-aspartic acid
Glycerol phenylbutyrate
N-acetylvaline
cis-Aconitate
CLONIXIN
Castanospermine
2-Methyl-4-propyl-1,3-oxathiane
